# Changes in Brain Electrical Activity after Transient Middle Cerebral Artery Occlusion in Rats

**DOI:** 10.3390/neurolint14030044

**Published:** 2022-06-21

**Authors:** Yuriy I. Sysoev, Veronika A. Prikhodko, Aleksandra V. Kan, Irina A. Titovich, Vadim E. Karev, Sergey V. Okovityi

**Affiliations:** 1 Department of Pharmacology and Clinical Pharmacology, Saint Petersburg State Chemical and Pharmaceutical University, 197022 Saint Petersburg, Russia; veronika.prihodko@pharminnotech.com (V.A.P.); kan.aleksandra@pharminnotech.com (A.V.K.); irina.titovich@pharminnotech.com (I.A.T.); sergey.okovity@pharminnotech.com (S.V.O.); 2 Laboratory of Neuroprosthetics, Institute of Translational Biomedicine, Saint Petersburg State University, 199034 Saint Petersburg, Russia; 3 I.P. Pavlov Institute of Physiology of the Russian Academy of Sciences, 199034 Saint Petersburg, Russia; 4 N.P. Bechtereva Institute of the Human Brain of the Russian Academy of Sciences, 197376 Saint Petersburg, Russia; 5 Pediatric Research and Clinical Center for Infectious Diseases of the Federal Medical Biological Agency, 197022 Saint Petersburg, Russia; vadimkarev@yandex.ru

**Keywords:** ischemic stroke, cerebral ischemia, brain electrical activity, electroencephalography, electrocorticography, rats

## Abstract

*Objectives.* Ischemic stroke is a leading cause of death and disability worldwide. To search for new therapeutic and pharmacotherapeutic strategies, numerous models of this disease have been proposed, the most popular being transient middle cerebral artery occlusion. Behavioral and sensorimotor testing, biochemical, and histological methods are traditionally used in conjunction with this model to assess the effectiveness of potential treatment options. Despite its wide overall popularity, electroencephalography/electrocorticography is quite rarely used in such studies. *Materials and methods.* In the present work, we explored the changes in brain electrical activity at days 3 and 7 after 30- and 45-min of transient middle cerebral artery occlusion in rats. *Results.* Cerebral ischemia altered the amplitude and spectral electrocorticogram characteristics, and led to a reorganization of inter- and intrahemispheric functional connections. Ischemia duration affected the severity as well as the nature of the observed changes. *Conclusions.* The dynamics of changes in brain electrical activity may indicate a spontaneous partial recovery of impaired cerebral functions at post-surgery day 7. Our results suggest that electrocorticography can be used successfully to assess the functional status of the brain following ischemic stroke in rats as well as to investigate the dynamics of functional recovery.

## 1. Introduction

Ischemic stroke (IS) is one of the leading causes of disability, and the second leading cause of death worldwide. Every year, IS affects millions of patients of all ages, ethnic and social groups [1]. Due to the neurological complications of cerebral ischemia, about 50% of IS survivors become chronically disabled [2]. Neuroprotective therapy, which is aimed at reducing brain damage and enhancing neuronal survival in the ischemic tissue, is a promising approach to the treatment of the neurological disorders resulting from IS. Despite the wide variety of proposed neuroprotective drugs, most of them have only been proven effective in animal studies [3]. Due to this, the search for and development of new neuroprotective agents is of utmost importance for the treatment of IS patients.

For the successful translation of animal research findings into clinical practice, experimental neuropharmacology requires objective methods to assess the effectiveness of investigational treatments. To date, in combination with rat models of transient middle cerebral artery occlusion (MCAO), most researchers employ behavioral and functional testing [4,5], biochemical [6,7], immunohistochemical [8,9] methods, and neuroimaging techniques, such as positron emission tomography (PET) [10] or magnetic resonance imaging (MRI) [11,12]. The listed approaches have their undoubted advantages and have proven themselves essential for experimental research; however, they all have certain disadvantages and limitations. For instance, behavioral and functional tests are quite subjective, biochemical and immunohistochemical studies usually require animal sacrifice, and PET and MRI are not available in most laboratories due to high cost.

Neurophysiological methods (electroencephalography (EEG), electrocorticography (ECoG)) are much less commonly employed to assess the neuroprotective activity of new drugs. The main difficulty that they pose to researchers is the need for surgical implantation of EEG/ECoG electrodes, which usually increases the time and labor intensity of the study, and also limits the number of experimental animals. In addition, the obtained neurophysiological data are complex and quite underexplored, and may therefore require relevant experience in order to be interpreted correctly. On the other hand, some aspects of the nervous system can only be studied with the help of neurophysiological methods. For instance, the degree of functional connectivity between different parts of the cerebral cortex (or underlying structures) can only be evaluated using ECoG or electrosubcorticography. The possibility of recording animal brain electrical activity (BEA) as often and for as long as necessary is another important advantage of neurophysiological methods.

Successful application of EEG/ECoG for evaluating the effectiveness of novel stroke treatments first of all requires a clear identification of the BEA parameters that are sensitive to cerebral ischemia in rodent MCAO models. In the future, they could serve as potential biomarkers of ischemia severity and, therefore, the effectiveness of neuroprotective therapy. In view of this, our study aimed to identify specific changes in the amplitude, spectral, and cross-correlation parameters of electrocorticograms in rats subjected to cerebral ischemia induced by transient MCAO.

## 2. Materials and Methods

### 2.1. Animals

The study was carried out in accordance with the principles of the Basel Declaration, Order of the Ministry of Health of the Russian Federation No. 199n (1 April 2016) ‘On the approval of the rules of good laboratory practice’, and the recommendations of the Bioethics Committee of Saint Petersburg State Chemical and Pharmaceutical University of the Ministry of Health of the Russian Federation. A total of 18 white outbred male rats weighing 250–300 g were obtained from the Rappolovo laboratory animal supplier (Leningrad Oblast, Russia). All animals were received in a single shipment, quarantined for 2 weeks, then housed in a standard animal facility with *ad libitum* access to standard chow (Laboratorkorm, Moscow, Russia) and drinking water. Prior to experimentation, the rats were randomized into 3 groups: Control (presumably healthy animals; *n* = 6), IS 30 (MCAO for 30 min; *n* = 6) and IS 45 (MCAO for 45 min; *n* = 6).

### 2.2. Surgical Procedures

Corticographic electrodes were prepared using 0.5 mm thick (active and reference) or 0.16 mm thick (ground) nichrome wire, heat-shrink insulated so as to leave a ~1 mm non-insulated conductive tip, and then assembled in a single-row, 8-pin, 2.54 mm pitch, straight female BLS-8 header connector.

The animals were anesthetized with 400 mg·kg^−1^ b.w. chloral hydrate (Sigma-Aldrich, Burlington, MA, USA) and treated with carbomer 974P gel (Ophthagel^®^, Santen OY, Tampere, Finland) to avoid corneal drying. During surgery, the animals were kept on an electric heating mat to keep body temperature within 37 ± 0.5 °C range. Following skin shaving and disinfection, a sagittal incision was made from the center of the occipital bone to the center of the frontal bone. To expose the cranial bone surface, the muscles, fascia, and periosteum were gently retracted and successively excised. Thermocoagulation hemostasis and hydration with 0.9% saline were provided as necessary. One millimeter deep cranial burr holes were drilled to implant the corticographic electrodes and fastening screws. Intermittent drilling and saline irrigation were used in order to avoid thermonecrosis due to excessive frictional heat generation.

The electrode implantation site coordinates were determined according to the Paxinos & Watson stereotaxic rat brain atlas [13]. Electrodes FP1 and FP2 were implanted above the secondary motor cortex (AP = +2.0, ML = 1.5, DV = 1.0), C3 and C4, the hindlimb primary motor cortex (AP = −1.0, ML = 2.0, DV = 1.0), and O1 and O2, the primary somatosensory cortex area, located above the hippocampal formation (AP = −4.0, ML = 2.0, DV = 1.0). The reference electrode was placed in the caudal part of the nasal bone, and the ground electrode was inserted subcutaneously in the neck region. The implants were secured and fixated on the cranial surface using Villacryl C dental acrylic resin (Zhermack, Badia Polesine, RO, Italy). The incision ends were then sutured, and the surrounding area was disinfected with iodine solution. Cerebral ischemia was inducted using the previously described intraluminal transient MCAO model [14]. The blood flow was blocked for 30 or 45 min (in groups IS 30 and IS 45, respectively); then the occluding filament was retracted, restoring blood supply in the MCA territory.

After surgery, the rats were kept in individual cages with *ad libitum* access to chow and water throughout the entire study period. Each animal’s condition was checked at recovery from anesthesia and then monitored twice a day, in the morning and in the evening. Suture disinfection with iodine solution was provided as necessary. In order to avoid dehydration, normal saline was injected subcutaneously for the first 3 days post-surgery. The use of antibiotics, analgesics, or anti-inflammatory drugs was consciously avoided, since most of those can affect the pathogenesis of ischemic stroke, possibly distorting the results of the study [15,16].

### 2.3. Electrocorticogram Acquisition and Analysis

Cortical electrical activity was recorded on post-surgery days 3 and 7 using an 8-channel Neuron-Spectrum-1 EEG system (Neurosoft, Ivanovo, Russia) at a 0.5–35 Hz bandwidth and a 500 Hz sampling rate. The two time points were chosen in order to assess the brain function of the injured animals in the subacute (day 3) and chronic (day 7) phases of IS [17,18]. Corticogram fragments of up to 5 min long which corresponded to awake, resting state with no locomotion, exploratory and/or grooming behavior were analyzed by Neuron-Spectrum.NETω software (Neurosoft, Ivanovo, Russia). Rats did not reach a stable sleep or immobility for a certain length of time before the registration period, testing was performed between 12.00 and 14.00. Artifacts were removed by visual inspection during offline analysis. After artifact rejection, signals were collected in 5-s units and fast Fourier transformed using a Hamming window. Next, amplitude, spectral, and cross-correlation analyses were carried out, and group means were compared for each parameter. For amplitude analysis, overall mean wave amplitudes were calculated. Spectral analysis included the calculation of mean wave amplitudes and rhythm indices for each of the δ (0.5–4.0 Hz), θ (4.0–8.0 Hz), α (8.0–14.0 Hz), and β frequency bands (low-frequency, LF-14.0–20.0 Hz, and high-frequency, HF-20.0–35.0 Hz). Rhythm indices were calculated as total duration percentages of signals registered in the δ, θ, α, and β frequency bands. Cross-correlation analysis included the calculation of cross-correlation coefficients (CCr) for electrode pairs FP1-C3, FP2-C4, C3-O1, C4-O2, FP1-FP2, C3-C4, and O1-O2.

### 2.4. Histological Examination

On day 7 following the completion of all experiments, the animals were euthanized by CO_2_ inhalation, and brains were extracted and fixed in 10% buffered formalin for 24 h. For histological analysis, the brain was cut coronally in 0.2–0.3 cm-thick sections at the ECoG electrode implantation level. Tissue samples were dehydrated, cleared in isopropanol, and embedded in paraffin according to standard protocols. Four µm sections were prepared from paraffin blocks using a HM340 rotary microtome (Fisher Scientific, Loughborough, UK), mounted on slides, stained with hematoxylin and eosin, dehydrated, and cover-slipped. Whole slide imaging was performed using a Pannoramic MIDI automatic digital slide scanner with Pannoramic Scanner Software for Research (3DHISTECH Kft, Budapest, Hungary).

### 2.5. Statistical Analysis

Statistical analysis of the data was performed using the Prism 7.00 software (GraphPad Software Inc., San Diego, CA, USA). The data were tested for normality using the Shapiro-Wilk W-test. Comparisons between groups (control, IS 30 and IS 45) were tested using the Kruskal-Wallis non-parametric test followed by Dunn’s *post hoc* test. The significance threshold was set at *p* < 0.05.

## 3. Results

**Figure 1:**
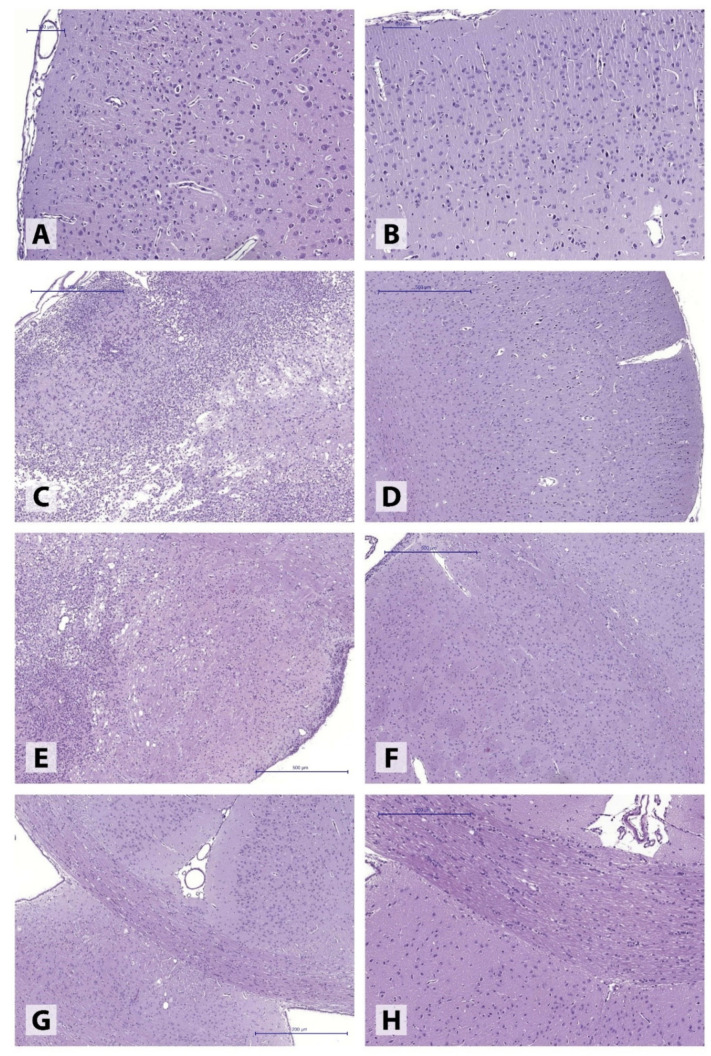
Histological features of different brain structures in experimental animals. (**A**,**B**) motor cortex ((**A**), right hemisphere; (**B**), left hemisphere) in an IS 45 rat; (**C**,**D**) somatosensory cortex ((**C**), right hemisphere; (**D**), left hemisphere) in an IS 45 rat; (**E**,**F**) striatum (**E**), right hemisphere, (**F**), left hemisphere in an IS 45 rat; (**G**,**H**) corpus callosum ((**G**) in an IS 45 rat; (**H**) in a control rat). Haematoxylin and eosin stain.

Histomorphological examination of brains obtained from IS 45 rats revealed extensive ischemic lesions (Figure 1) covering large portions of somatosensory cortex and spreading into deep basal ganglia structures, reaching the subventricular zone (Figure 1C,E). The lesions were characterized by large necrotic foci accompanied by ongoing liquefaction, prominent perifocal histiocytic reaction (predominantly featuring numerous microglial cells), and extensive polymorphonuclear leukocyte infiltration. The adjacent brain tissue was characterized by extensive, uneven perivascular and pericellular edema as well as cell dystrophy of varying degrees. At the same time, the motor cortex (Figure 1A) and corpus callosum (Figure 1G) areas remained intact.

**Figure 2:**
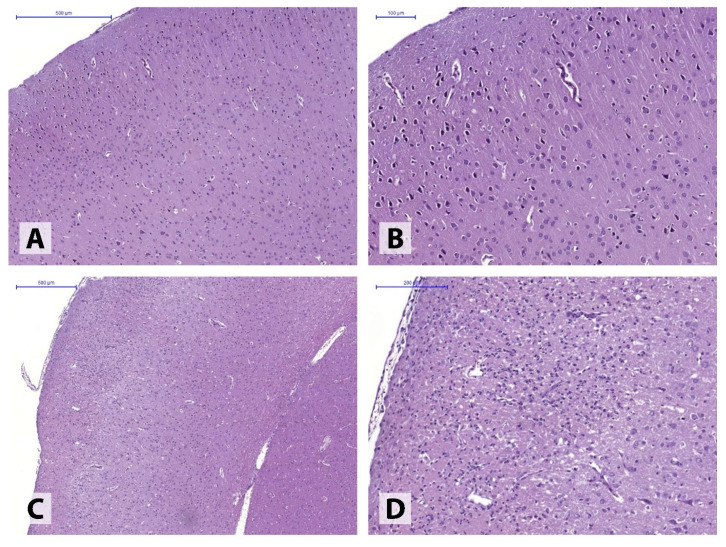
Minimal (**A**,**B**) and severe (**C**,**D**) pathological alterations in the somatosensory cortex following 30-min ipsilateral ischemia. Haematoxylin and eosin stain.

The ischemic hemisphere following 30-min MCAO presented with markedly polymorphic pathological alterations in different brain structures. In the majority of the animals (4), perivascular and pericellular edema of varying degrees was observed, accompanied by hypoxia-induced neuronal dystrophic changes, without the loss of normal tissue structure (Figure 2A,B). In some animals (2), the post-ischemic alterations involved the formation of extensive tissue necroses in the injured hemisphere, found in both motor and somatosensory cortex (Figure 2C,D).

**Figure 3:**
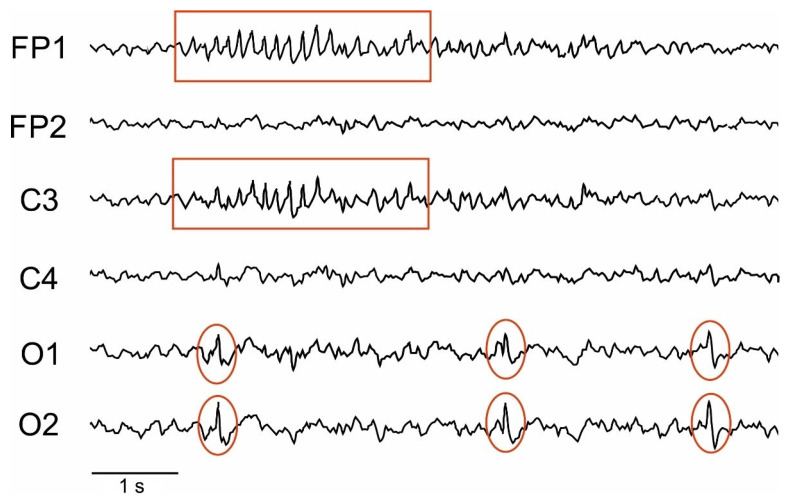
Epileptic activity (within red frames) observed in an IS 45 rat at post-surgery day 7. Red ellipses mark the spikes in the O1/O2 area (above the hippocampus).

Our study found that transient MCAO-induced cerebral ischemia in rats affected the amplitude, spectral, and cross-correlation parameters of BEA (Tables S1 and S2). The nature and severity of the observed changes were dependent on ischemia duration as well as the time after ischemia induction. In one of the IS 45 rats, periodic epileptic activity (approximately every 12 s) in the FP1/C3 area of the non-ischemic hemisphere was recorded on post-surgery day 7. Additionally, the rat developed periodic spike activity in the O1/O2 area, above the hippocampus (Figure 3). Recording fragments containing spikes or sharp waves were omitted from further analysis.

**Figure 4:**
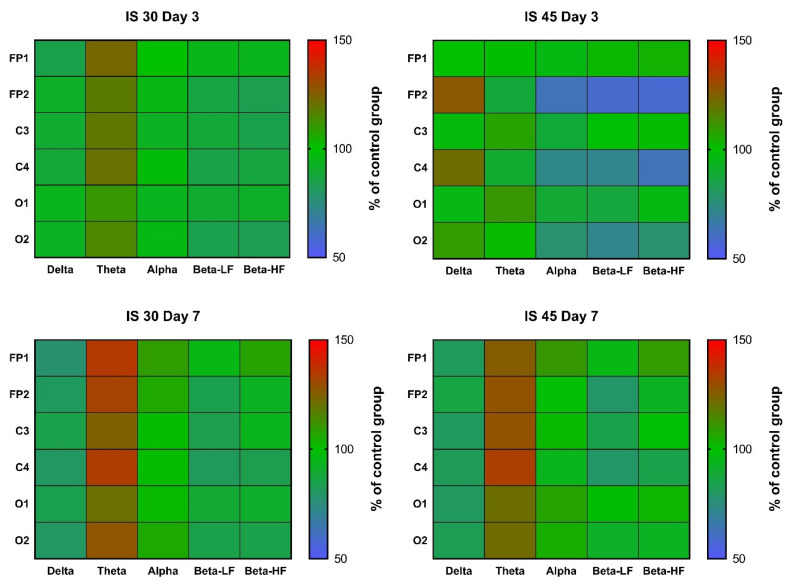
Heatmaps of δ, θ, α, high and low frequency β wave rhythm indices in channels FP1, FP2, C3, C4, O1, and O2 at post-surgery days 3 and 7. Data were normalized to the respective control values (*n* = 6). IS 30 (*n* = 6) and IS 45 (*n* = 6), groups subjected to cerebral ischemia for 30 and 45 min, respectively.

In rats subjected to 30-min MCAO, an increase in θ rhythm indices in channels FP1 and C3 was observed at post-surgery day 3 (*p* < 0.05 vs. Control for both). A similar increase was seen in all other channels, but did not reach statistical significance (Figure 4, Table S1). In the IS 45 group, a decrease in α, β-LF and β-HF rhythms (*p* < 0.05 for channel FP2 in all cases) was accompanied by a slight increase in δ rhythm (not statistically significant) in the ischemic hemisphere (Figure 4, Table S1). At post-surgery day 7, the changes were the same: a non-specific decrease in δ rhythm indices (not statistically significant) and an increase in θ rhythm (IS 30: *p* < 0.01 for channel C4, *p* < 0.05 for channels FP1, FP2, and O2; IS 45: *p* < 0.01 for channel C4, *p* < 0.05 for channels FP2 and C3) (Figure 4, Table S2).

**Figure 5:**
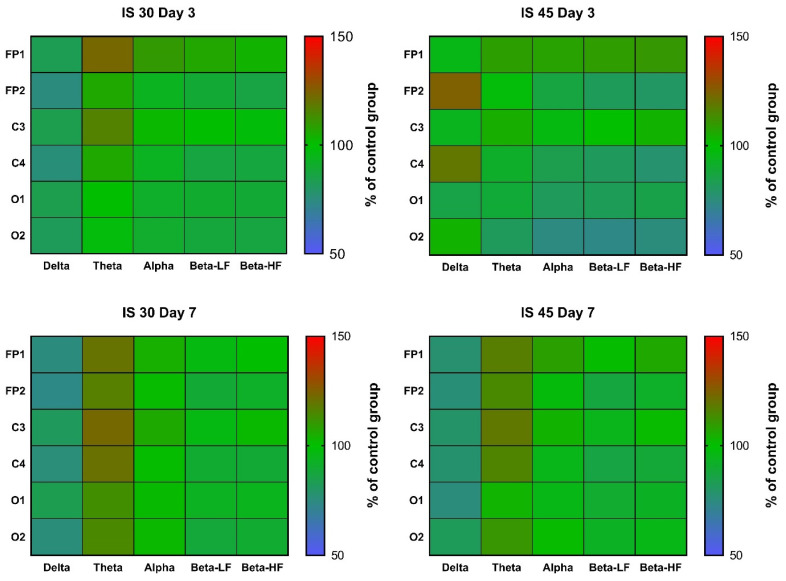
Heatmaps of mean amplitudes of δ, θ, α, high and low frequency β waves in channels FP1, FP2, C3, C4, O1, and O2 at post-surgery days 3 and 7. Data were normalized to the respective control values (*n* = 6). IS 30 (*n* = 6) and IS 45 (*n* = 6), groups subjected to cerebral ischemia for 30 and 45 min, respectively.

Mean wave amplitudes were not significantly different among all groups both on post-surgery days 3 and 7 (Figure 5, Tables S1 and S2). Notably, the changes in these parameters resembled the changes in rhythm indices mentioned earlier; for instance, we observed reduced δ activity in the FP2/C4 area in the IS 45 group on day 3, as well as a non-specific decrease in δ accompanied by an increase in θ activity in both IS groups on day 7 (Figure 5, Tables S1 and S2).

**Figure 6:**
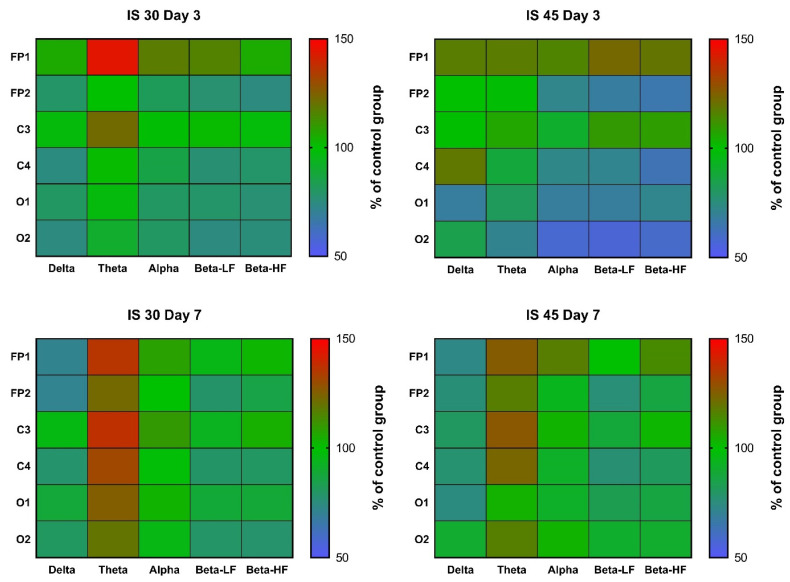
Heatmaps of mean power of δ, θ, α, high and low frequency β waves in channels FP1, FP2, C3, C4, O1, and O2 at post-surgery days 3 and 7. Data were normalized to the respective control values (*n* = 6). IS 30 (*n* = 6) and IS 45 (*n* = 6), groups subjected to cerebral ischemia for 30 and 45 min, respectively.

Spectral analysis did not reveal any distinct patterns of changes in any of the IS groups post-surgery day 3 (Figure 6, Table S1). There was a slight decrease in α and β activity, which was more pronounced in IS 45 rats; however, due to the high data variability in all groups, no statistically significant differences were observed (Figure 6, Table S1). At day 7, the pattern of changes resembled that of the changes in rhythm indices and mean wave amplitudes; namely, a decrease in δ and an increase in θ activity were seen (not statistically significant) (Figure 6, Table S2).

**Figure 7:**
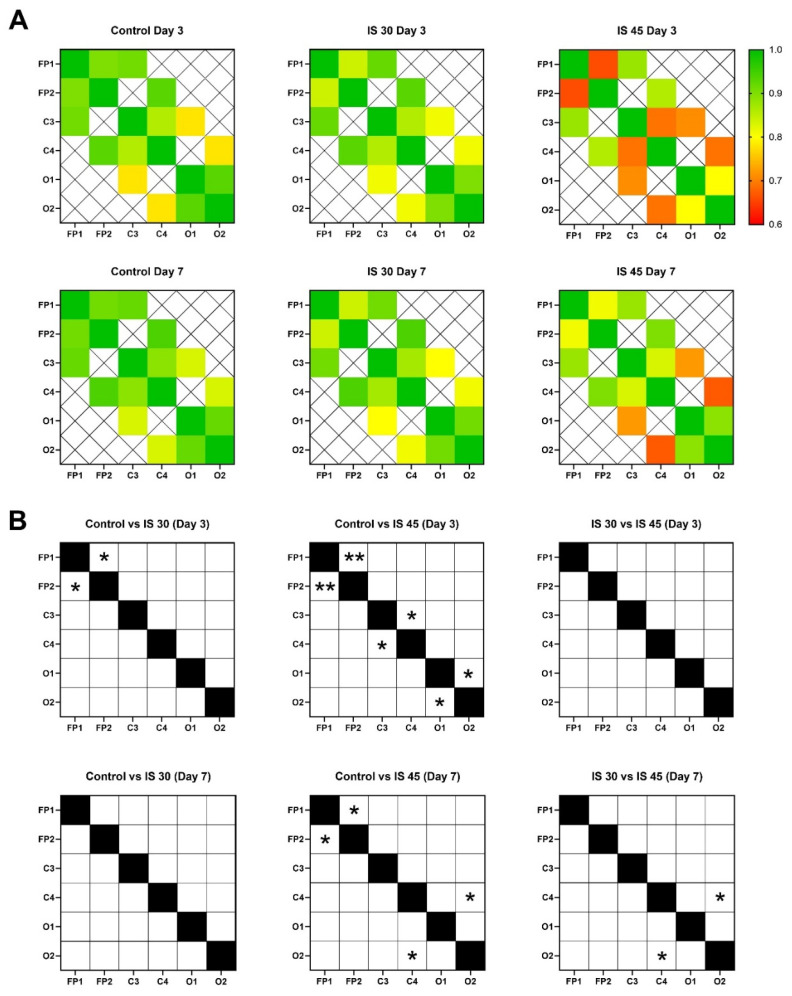
Cross-correlation matrices (**A**) and statistical analysis results (**B**) for pairwise correlations FP1-FP2, C3-C4, O1-O2, FP1-C3, FP-C4, C3-O1, and C4-O2 for all groups. Connectivity strength is color-coded according to (**A**). Empty cells in (**B**) indicate no significant difference among the Control and IS 30 or IS 45 groups, whereas cells with * or ** indicate significant differences among groups. **, *p* < 0.01; *, *p* < 0.05.

Another important feature of ischemic brain injury in both IS 30 and IS 45 groups was a disruption of inter- and intrahemispheric functional connectivity, as evidenced by decreased Ccr values for different electrode pairs (Figure 7, Tables S1 and S2). For example, in IS 30 animals, a statistically significant (*p* < 0.05 vs. Control) decrease in Ccr for electrode pair FP1-FP1 was seen at post-surgery day 3. On day 7, no significant differences were found for IS 30 vs. Control. Quite unsurprisingly, longer ischemic exposure led to a more profound disruption of inter- and intrahemispheric functional connectivity. Compared to Control, IS 45 rats had lower Ccr for pairs FP1-FP2 (*p* < 0.01), C3-C4 (*p* < 0.05), and O1-O2 (*p* < 0.05) at post-surgery day 3, and for pairs FP1-FP2 (*p* < 0.05) and C4-O2 (*p* < 0.05) at day 7. No statistically significant differences were found between the two IS groups at day 3; however, at day 7, IS 45 had a lower Ccr value for channel pair C4-O2 than both the Control and IS 30 groups (*p* < 0.05).

## 4. Discussion

The present study explored the most specific changes in BEA in the subacute (3 days) and chronic (7 days) phases of IS induced by 30- and 45-min MCAO. The choice of the study timepoints was due to two main reasons. First, the main pathological processes leading to neuronal loss, such as excitotoxicity, oxidative stress, and apoptosis occur in the first few days or up to a week after a stroke in rats [19,20]. As a result, most experimental research of novel neuroprotective agents is focused on those periods [21]. Second, similar timepoints were chosen in some of the previous studies using a rat model of traumatic brain injury (TBI) [22,23]. Unilateral TBI was shown to cause specific changes in the amplitude, spectral, and cross-correlation parameters of rat electroencephalograms; at the same time, a positive trend towards recovery was observed at post-TBI day 7 compared with day 3. Since traumatic and vascular brain injuries are quite similar in pathogenesis and dynamics [24,25], we have decided to observe the already established experimental protocol. In addition, this allowed us to compare the electrophysiological features of two commonly used models of central nervous system injuries: transient MCAO (IS) and controlled cortical impact (TBI). Certainly, electroencephalography use after IS or TBI is not limited to 7 days, and it can also be employed to study the long-term consequences of vascular and traumatic brain injuries in rodents.

An increase in δ, and a decrease in α and β rhythms in the ischemic hemisphere in rats subjected to 45-min MCAO were an expected outcome, since the same changes were observed in a rat model of TBI at post-surgery day 3 [26]. Thus, the nature of changes in the spectral parameters of BEA can be considered specific for the subacute phase of organic brain injuries. Changes in other amplitude and spectral parameters (mean rhythm amplitudes and mean power) generally followed the same pattern in the subacute phase after 45-min MCAO, although in a less pronounced way. This similarity is quite understandable, since all three parameters are closely interrelated, and an increase in δ rhythm index seems unlikely in the absence of at least some increase in its mean amplitude. Nevertheless, in our previous work [26], mean rhythm amplitudes were shown to represent the general pattern of ongoing processes in the brain during traumatic brain injury (a decrease in the mean amplitudes of θ-, α-, and β-rhythms, regardless of the lead). For the rhythm indices, distinct changes were observed in the injured hemisphere (leads FP1, C3, and O1), while in the healthy hemisphere the changes were mild, and it could be concluded that they almost do not occur, if at all, in the absence of any pathological changes. Therefore, in this study, we focused on the changes in the three parameters mentioned above, however unfortunately, unlike the case with TBI, we did not observe different heatmap patterns here.

In our study, the key change in the amplitude and spectral electrocorticogram characteristics in IS was represented by the dynamics of the θ rhythm. In general, its increase (accompanied by a slight decrease in δ activity) in all channels was characteristic of IS 30 rats at post-surgery day 3, as well as of both IS groups on day 7. This may indicate that IS 45 animals reached partial functional recovery on day 7 after IS, paralleling the functional status of the IS 30 group. Nevertheless, the shift in the signal spectrum towards the θ rhythm remained the same in IS 30 rats throughout both the subacute and chronic phases. These data suggest that a non-specific increase in θ activity is an indicator of ischemic brain damage, which is independent of whether the ischemia was focal or affected the entire brain. In addition, this indicator seems stable enough over time, but further research is needed to determine for how long θ activity remains increased after cerebral ischemia.

In a similar study, Zhang et al. [27] explored the dynamics of rat BEA in the acute (3–6 h), subacute (12–48 h), and chronic (72–168 h) periods after 90-min MCAO. The ischemic hemisphere exhibited decreased α and β activity at post-surgery day 3, as well as increased θ activity at day 7 (compared with day 3). In addition, a steady rise in δ rhythm was recorded for up to 72 h after MCAO. The authors note that the absolute values of δ, α, and β rhythms as well as the α/δ rhythm ratio correlated with the De Ryck scores and performance in the beam walking test. In contrast to the present study, the authors dismissed the marked and stable increase in θ activity, although it was observed on spectral power graphs starting from 96 h after ischemia induction.

A decrease in the Ccr values of certain electrode pairs following IS indicates a disruption of both inter- and intrahemispheric functional connections. Similar to the amplitude and spectral parameters, we observed a positive dynamic of brain connectivity in IS animals at post-surgery day 3. This fact appears to be yet another similarity to the changes in BEA described previously in a rat model of TBI [26]. It is important to note that Ccr were also sensitive to ischemia severity, and further decreased when MCAO was prolonged from 30 to 45 min.

Although in clinical practice, EEG is most commonly used to monitor for epileptiform activity, it can also be used for intraoperative detection of early mild ischemic damage, e.g., during carotid artery surgery [28]. In addition, in patients with anterior cerebral artery territory infarcts, the Brain symmetry index values (representing the differences in mean signal spectral powers in the 1–25 Hz band between the two hemispheres) are highly correlated with the National Institutes of Health stroke scale (NIHSS) scores [29]. Another study [30] showed that acute delta change index (aDCI) values have a stronger correlation with NIHSS scores 30 days after IS than do infarct volumes seen on diffusion-weighted MRI in the early phase of stroke. Spectral Exponent (SE), a metric that reflects EEG slowing and quantifies the power-law decay of the EEG Power Spectral Density was shown to be sensitive to MCA stroke in patients and demonstrated renormalization after two months of physical rehabilitation. It is worth noting that SE renormalization significantly correlated with NIHSS improvement [31]. Another important measure is the slope of the power spectrum (also referred to as 1/f noise) may be potentially useful in clinical practice in patients with stroke. Since rat data indicate that 1/f is highly correlated with poststroke motor function [32], this measure may be successfully applied for human stroke EEG data. These examples strongly support the possibility of using EEG as a means of predicting the severity of delayed neurological disorders in the acute or chronic phase of cerebral ischemia. In general, BEA analysis is a sensitive tool for assessing the functional status of the brain in both animals and humans. In view of this, the development of new approaches to EEG/ECoG analysis in small laboratory animals, and their subsequent use in stroke patients is a priority for current research.

It should be noted that in this paper, we focused on the use of ECoG for experiments in rodents. The observed changes in the BEA are specific to rats (and most likely, to other rodents), but may not be relevant for humans. This may be due to the fact that rats and humans may have different ranges of EEG rhythms; for example, it was shown that rodent θ (6–10 Hz) is an overlapping but overall faster frequency range than human θ (4–7 Hz) [33]. In addition, electrode characteristics (e.g., material, size), reference electrode location, recording device parameters, and other methodological factors play an essential role in the amplitude-spectral characteristics of ECoG in rats. Despite the fact that attempts are being made to standardize EEG studies in rodents [34], the obtained results often do not correlate not only with data from humans, but also among rodent studies performed by different laboratories. Nevertheless, if, in the future, the method we have described could indicate the ability of a potential neuroprotective agent to ameliorate pathological BEA changes in experimental stroke, as shown earlier for TBI [26], it would possibly predict positive effects of said agent in human patients as well.

## 5. Conclusions

The present study demonstrated that IS significantly alters BEA, which can be observed in the subacute (day 3) and chronic (day 7) phases after transient MCAO in rats. The nature and severity of these alterations can change over time, indicating partial functional recovery of the brain. Our results suggest that the registration and analysis of ECoG is a viable tool to be used in combination with rat MCAO models for evaluating the effectiveness of novel therapeutic and pharmacotherapeutic approaches to the treatment of neurological disorders.

## Data Availability

The main data are available with the corresponding author.

## Supplementary Materials

The following supporting information can be downloaded at: https://www.mdpi.com/article/10.3390/neurolint14030044/s1, Table S1: Day 3, Table S2: Day 7.
